# Red blood cell distribution width as a prognostic marker in patients with heart failure and diabetes mellitus

**DOI:** 10.1186/s12933-017-0563-1

**Published:** 2017-07-06

**Authors:** Andrew Xanthopoulos, Gregory Giamouzis, Andreas Melidonis, Takeshi Kitai, Efi Paraskevopoulou, Pinelopi Paraskevopoulou, Sotirios Patsilinakos, Filippos Triposkiadis, John Skoularigis

**Affiliations:** 1 Department of Cardiology, University General Hospital of Larissa, P.O. Box 1425, 411 10 Larissa, Greece; 2 Department of Internal Medicine, Tzaneio Hospital, Athens, Greece; 3 Department of Cardiovascular Medicine, Kobe City Medical Center General Hospital, Kobe, Japan; 4 Department of Cardiology, Konstantopoulio General Hospital, Athens, Greece; 5 Hematology Lab, Konstantopoulio General Hospital, Athens, Greece

**Keywords:** RDW, Heart failure, Diabetes mellitus, Prognosis

## Abstract

**Background:**

Red blood cell distribution width (RDW) is an established prognostic marker in acute and chronic heart failure (HF). Recent studies have pointed out a link among RDW, diabetes mellitus (DM) and inflammation. We sought to investigate the prognostic value and longitudinal pattern of RDW in patients with concomitant HF and DM, which remains unknown.

**Methods:**

A total of 218 patients (71 diabetics) who presented with acute HF had RDW measured at admission, discharge and 4, 8 and 12 months post-discharge. The study endpoint was all-cause mortality or rehospitalization for HF during 1-year follow-up.

**Results:**

The study endpoint was met in 33 patients (46.5%) with DM and in 54 patients (36.7%) without DM. RDW at admission was associated with higher event rate both in HF patients with and without DM (adjusted HR: 1.349, p = 0.002, 95% CI 1.120–1.624 and adjusted HR: 1.142, p = 0.033, 95% CI 1.011–1.291 respectively). In addition, a significant interaction was found between diabetes and RDW longitudinal changes (β_interaction_ = −0.002; SE = 0.001; p = 0.042).

**Conclusions:**

Despite the similar prognostic significance of RDW in diabetic and non-diabetic HF patients regarding the study endpoint, longitudinal changes were found to be significantly different between these two groups of HF patients. This might be due to the higher inflammatory burden that diabetic HF patients carry and may provide new insights to the pathophysiological mechanism of RDW increase in HF, which remains unknown.

## Introduction

Red blood cell distribution width (RDW) is a simple parameter of the standard full blood count and a measure of heterogeneity in the size of circulating erythrocytes. It is provided by automated hematology analyzers and it reflects the range of the red cell size [1]. It is calculated by dividing the standard deviation of erythrocyte volume by the mean corpuscular volume (MCV) and multiplied by 100 to convert to a percentage [2]. Although RDW has been traditionally used in the investigation of the etiology of anemia [3] there is an increasing evidence linking elevated RDW with poor outcome in general population [4, 5], in patients with coronary artery disease [6–9], with metabolic syndrome [10] and heart failure (HF) [11–19]. Importantly, this association was independent of other hematological variables such as MCV, hemoglobin and hematocrit levels. Diabetes mellitus (DM) is a common comorbidity and an independent risk factor for HF [20]. An increase in HbA1c of 1% correlates to an increment of 8% in HF [21]. In addition, diabetic patients have an almost twofold increased risk of HF [22], while the prevalence of DM in HF patients varies from 19 to 31% [23, 24].Thus, not surprisingly, HF has been termed “the frequent, forgotten, and often fatal complication of diabetes” [25]. The term “diabetic cardiomyopathy” was initially introduced based upon postmortem findings in four diabetic adults who had HF in the absence of coronary artery disease and has been defined as ventricular dysfunction that occurs in diabetic patients independent of a recognized cause (e.g. coronary artery disease, hypertension) [26–28]. Recent studies demonstrate an interesting interaction between diabetic status and RDW [29, 30] and consider the latter as “an inflammatory marker with a significant predictive value of mortality in diseased and healthy populations”. Studies of RDW in patients with HF, either in the acute or chronic setting, and concomitant DM are lacking [31]. Therefore, the aim of this exploratory study was to investigate the longitudinal changes and prognostic value of RDW in diabetic patients initially admitted for acute heart failure (AHF) and to compare these findings with a control group (AHF patients without DM).

## Methods

We conducted a prospective cohort study on the Cardiology Departments of two General Hospitals. Patient’s recruitment began in January 2015 and completed in July 2015. The follow up period (at 4, 8 and 12 months post discharge) lasted 1 year. Surveillance was carried out in 218 consecutive patients (60.6% males) who presented in the emergency department with symptoms of AHF (decompensated chronic HF or de novo HF). Among them 32.6% were diabetic. The AHF was defined as rapid or gradual onset of signs and symptoms of worsening HF resulting in unplanned hospitalization [32]. Diabetic status was defined by the presence of antidiabetic treatment in the patients’ medical history. Patients with Hemoglobin <10 g/dL and those who had received blood transfusions, Ferrum, B12, folic acid or had active cancer were excluded from the study.

The baseline evaluation of the patients included clinical assessment (medical history—cardiovascular risk factors, weight measure, New York Heart Association classification), laboratory blood tests and echocardiographic investigation. Levels of hematocrit, RDW, platelets (PLT) and white blood cells (WBC) were measured with the use of the Siemens Advia 2120 (Siemens Healthcare Diagnostics, INC, Deerfield, IL, USA) on samples obtained for standard of care evaluation. The normal reference range for RDW in the hospital laboratories that participated in the study was 11.5–15% with intra-assay variation of 2.38% and inter-assay variation of 1%. Glucose, urea, creatinine, SGOT, SGPT, electrolytes and uric acid levels were measured with Siemens Dimension (Siemens Healthcare Diagnostics, INC, Deerfield, IL, USA). Lastly, ferritin, folic acid, B12 and erythropoietin (EPO) levels were measured with the use of Access 2 Leriva (Leriva Diagnostics, SA, Beckman Coulter Immunoassay Systems, USA) and HbA1C with the use of EXL Siemens (Siemens Healthcare Diagnostics, INC, Deerfield, IL, USA). The echocardiographic examination was performed with the use of General Electric Vivid 7 machine (GE Healthcare, Horten, Norway) by two independent echocardiographers. Having in mind that the average lifespan of the red blood cells (RBC’s) is 120 days, the clinical, laboratory and echocardiographic evaluation were planned to be undertaken during the initial hospitalization (admission and discharge) and the pre-scheduled follow up (4, 8 and 12 months).

All patients during hospitalization and at discharge were under full up to date guideline directed medical therapy, unless there was a side effect or a contraindication in a specific drug category. The endpoint of the study was death from any cause or rehospitalization for HF (whichever occurred first).

### Statistical analysis

Continuous data are presented as medians with inter-quartile ranges for non-normally distributed variables and means ± standard deviations (SD) for all normally distributed variables. Categorical data are presented with absolute and relative (%) frequencies. To estimate the prognostic value of RDW in diabetic and non-diabetic patients with HF a Cox Regression analysis was used. We constructed multivariable Cox Proportional Hazard models with the risk adjusting variables listed in Table 1 to estimate the adjusted hazard ratios (HRs) and 95% confidence intervals (CI). Furthermore, the interaction between the RDW and DM was investigated just to examine whether the prognostic value of RDW differs between diabetics and non-diabetics, with the use of multivariate Cox Regression Analysis.

**Table 1:** Baseline patient characteristics according to diabetes status

Variables	Total (N = 218)	Diabetes (N = 71)	No diabetes (N = 147)	p value
Age (years), mean (SD)	73.9 (11.5)	73 (11.2)	74.4 (11.7)	0.399
Male gender, N (%)	132 (60.6)	45 (63.4)	87 (59.2)	0.658
Systolic blood pressure (mmHg), median [IQR]	155 [30]	160 [30]	155 [28]	0.837
Diastolic blood pressure (mmHg), median [IQR]	95 [10]	95 [10]	95 [13]	0.983
Heart rate (bpm), mean (SD)	116.2 (13.2)	113.5 (12.9)	117.9 (13.2)	0.334
HFrEF, N (%)^b^	157 (72)	48 (67.6)	109 (74.1)	0.313
EF (%), median [IQR]	37 [25]	37 [25]	37 [25]	0.127
Hypertension, N (%)	144 (66.1)	49 (69)	95 (64.6)	0.521
Atrial fibrillation, N (%)	109 (50)	33 (46.5)	76 (51.7)	0.411
Coronary artery disease, N (%)	75 (34.4)	26 (36.6)	49 (33.3)	0.229
NYHA class (admission)				
II	4 (1.8)	2 (2.8)	2 (1.4)	0.646
III	58 (26.6)	17 (23.9)	41 (27.9)	
IV	156 (71.6)	52 (73.2)	104 (70.7)	
B-blocker^a^, N (%)	181 (83)	60 (84.5)	121 (82.3)	0.686
ACE-inhibitor^a^, N (%)	138 (63.3)	40 (56.3)	98 (66.7)	0.138
Loop diuretics^a^, N (%)	193 (88.5)	63 (88.7)	130 (88.4)	0.949
MRA’s^a^, N (%)	132 (60.6)	42 (59.2)	90 (61.2)	0.77
Metformin^a^, N (%)	60 (27.5)	60 (84.5)	–	–
Sulfonylurea analogues^a^, N (%)	11 (5)	11 (15.5)	–	–
Insulin^a^, N (%)	22 (10.1)	22 (30.9)	–	–
Hematocrit (%), mean (SD)	40.4 (4.6)	40.1 (5)	40.6 (4.3)	0.874
RDW (%), mean (SD)	15.2 (1.6)	15.2 (1.5)	15.3 (1.7)	0.744
K^+^ (mmol/L), mean (SD)	4.6 (0.8)	4.6 (0.8)	4.6 (0.7)	0.718
Uric acid (mg/dL), mean (SD)	7.2 (2.2)	7.1 (2)	7.3 (2.3)	0.841
Ferritin (ng/mL), median [IQR]	55 [72]	72 [82.1]	52 [59.5]	*0.043*
B12 (pg/mL), median [IQR]	257 [239]	258 [208]	250 [256]	0.660
Glucose (mg/dL), median [IQR]	133 [70]	182 [127]	116.5 [38]	*<0.0001*
HbA1C (%), median [IQR]	6.3 [1.4]	7.6 [0.6]	6.1 [0.5]	*<0.0001*
Urea (mg/dl), median [IQR]	56 [35]	58 [40]	54 [33]	0.652
EPO (mIU/mL), median [IQR]	13.5 [13.4]	14.6 [13.9]	12.5 [13.1]	0.156
Creatinine (mg/dL), median [IQR]	1.2 [0.7]	1.2 [0.7]	1.1 [0.6]	0.231
SGOT (IU/L), median [IQR]	27 [21]	23 [19]	27.5 [20]	0.263
SGPT (IU/L), median [IQR]	34 [23]	35 [18]	32.5 [29]	0.965
Na^+^(mmol/L), median [IQR]	140 [7]	138 [7]	141 [6]	*0.015*
WBC (10^3^/mm^3^), median [IQR]	8.8 [4.2]	10.1 [4.6]	8.3 [4.4]	*0.012*
Folic Acid (ng/mL), median [IQR]	7 [4.2]	7.6 [4.6]	6.5 [3.7]	*0.027*
Weight (kg), median [IQR]	78 [25]	82 [27]	76 [21]	*0.04*

^a^Monotherapy or combination therapy

^b^HFrEF: EF < 50%

The results were confirmed using the ROC analysis and the estimation of the area under the curve (AUC), which values ranged from 0 to 1. RDW was considered to be better prognostic marker the closer the value of AUC was to 1. To investigate the longitudinal changes of the RDW in diabetic and non-diabetic patients a Linear Mixed Model was used, where diabetes and time as well as their interaction was modeled as fixed effects and patients as a random effect. Linear mixed models are used in longitudinal studies as they exhibit several advantages against standard classical models [33, 34]. All the variables in Table 1 were included in this model. Differences were considered significant when p < 0.05. For statistical analyses, SPSS 20.0 (SPSS Inc. Chicago, IL, USA) and STATA v.13 softwares were used.

## Results

The study endpoint was met in 33 (46.5%) HF patients with DM (mortality:21.1%, rehospitalization:25.4%) and in 54 patients (36.7%) without DM (mortality:15.6%, rehospitalization:21.1%).

Baseline characteristics of HF patients divided to diabetic and non-diabetic group are presented in Table 1. Patients with DM had significantly higher ferritin and WBC values compared to those without DM. Furthermore, diabetics showed higher folic acid, HbA1C and glucose values and they were more obese against the group of patients without DM. On the contrary, the latter manifested significantly higher sodium (Na^+^) levels. No statistically significant difference was observed with regard to RDW, platelets (PLT), renal or hepatic function, ejection fraction (EF), B12 and EPO.

With respect to antidiabetic therapy, the vast majority of DM patients were receiving metformin (mean dose 1211.25 ± 348.4 mg), followed by sulfonylurea analogues and insulin (Table 1).

### RDW as a prognostic marker in diabetic and non-diabetic patients with HF

Cox regression analysis revealed a statistically significant association between the RDW values at admission (adjusted HR: 1.349, p = 0.002, 95% CI 1.120–1.624) in the diabetic group and the study endpoint (all-cause mortality or rehospitalization for HF), adjusted for the variables listed in Table 1. In particular, 1% increase of the RDW value at admission corresponded to 34.9% increased hazard probability of experiencing the primary endpoint. Similarly, RDW at admission was of prognostic significance in non-diabetics (adjusted HR: 1.142, p = 0.033, 95% CI 1.011–1.291) (Table 2).

**Table 2:** Multivariate Cox Regression Analysis for the primary study endpoint (death or rehospitalization) in diabetic and non-diabetic heart failure patients

Variables	Hazard ratios	95% CI	p value
Diabetics			
RDW	1.349	1.120–1.624	0.002
Hypertension	0.426	0.210–0.862	0.018
Coronary artery disease	3.107	1.537–6.278	0.002
Non-diabetics			
RDW	1.142	1.011–1.291	0.033
Hypertension	0.229	0.125–0.417	<0.0001
Coronary artery disease	2.275	1.316–3.933	0.003

**Fig. 1:**
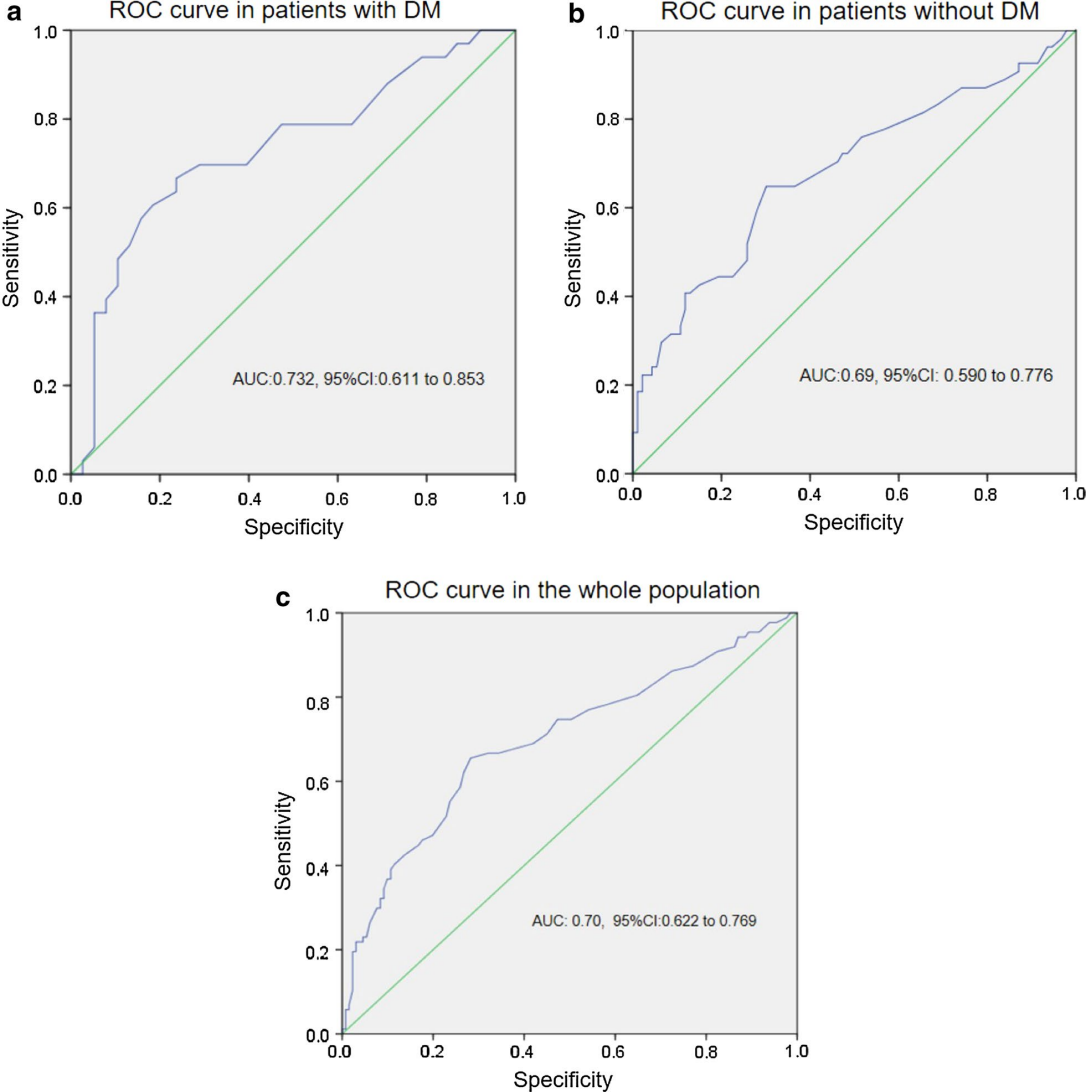
**a** Receiver operating characteristics (ROC) curve for the RDW at admission in patients with HF and DM. **b** Receiver operating characteristics (ROC) curve for the RDW at admission in patients with HF and without DM. **c** Receiver operating characteristics (ROC) curve for the RDW at admission in the whole population (HF patients with and without DM)

No statistically significant interaction was detected between RDW at admission and DM, in the Cox Regression analysis, indicating that the marker—at admission—is similarly associated with the primary outcome (all cause death or rehospitalization for HF) in both diabetics and non-diabetics (p-for interaction = 0.936). In conclusion, RDW was of prognostic importance with respect to the outcome (death or rehospitalization) in HF patients (diabetic or not). The previous results were also confirmed by the ROC analysis and the area under the curves (AUCs) in diabetic and non-diabetic HF patients (AUC: 0.732, 95% CI 0.611–0.853 and AUC: 0.69, 95% CI 0.590–0.776) for the study endpoint, which showed that RDW was of significant prognostic value in both groups. Similarly, the ROC analysis in the whole population of the study revealed an AUC: 0.70, 95% CI (0.622–0.769) (Fig. 1).

### Longitudinal changes of RDW in diabetic and non-diabetic patients with HF

**Fig. 2:**
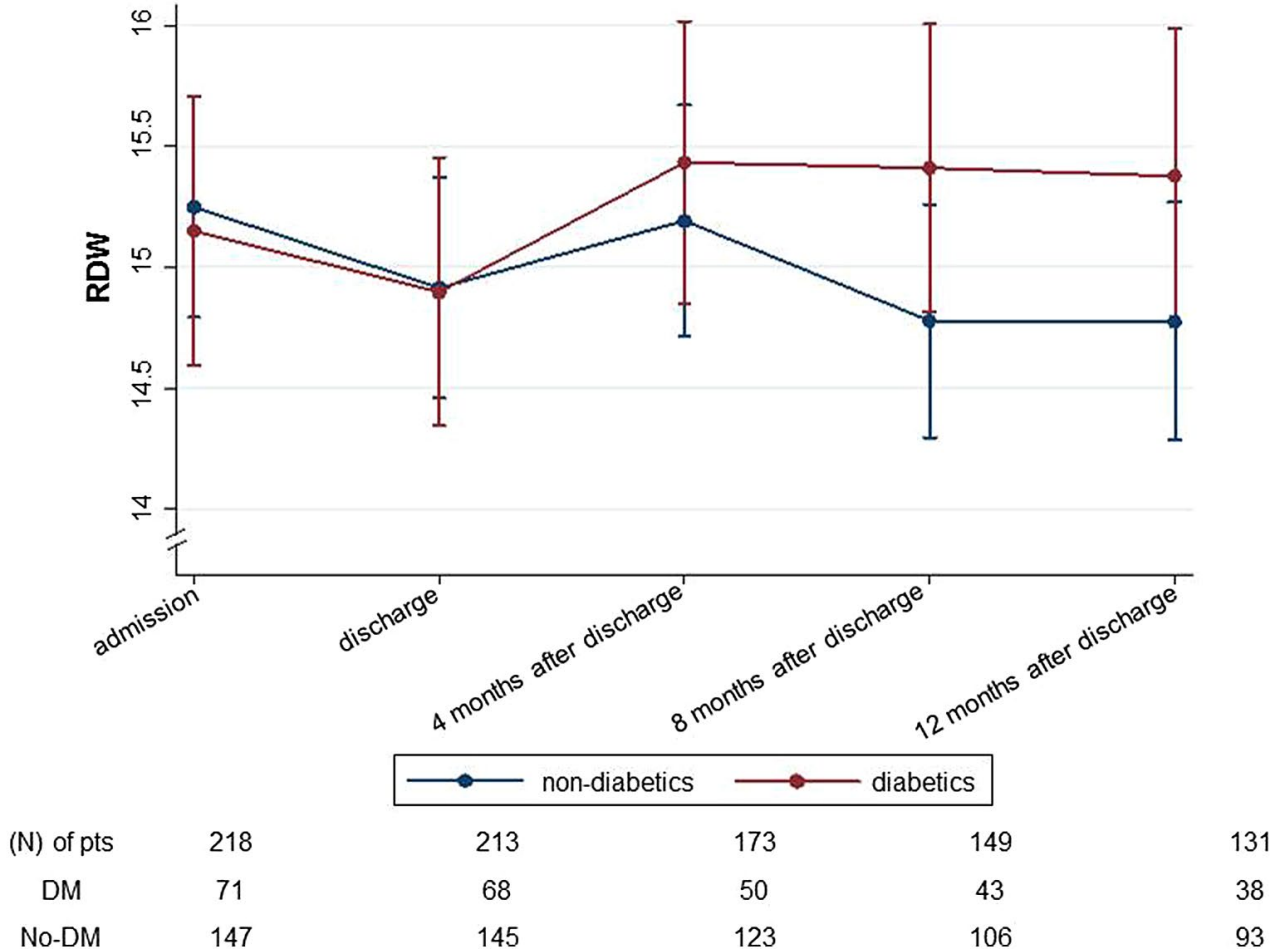
Repeated measures of RDW in diabetics and non-diabetics

To examine the longitudinal changes of RDW in diabetic and non-diabetic patients with HF, Linear Mixed Models were used. The latter showed that there is a statistically significant interaction (β_interaction_ = −0.002; SE = 0.001; p = 0.042) between DM and RDW longitudinal changes from the patient’s admission to 1-year post discharge. The other significant and independent factors were Hematocrit, EF and ACE-inhibitors (p < 0.001; p = 0.001; p = 0.033, respectively). Accordingly, the RDW longitudinal changes differed between the diabetic and non-diabetic HF patients. In other words; the estimating change of RDW values over 5 repeated assessments at the individual level, between the two groups of patients (diabetics and non-diabetics) was significantly different (Fig. 2).

## Discussion

Our study demonstrated that RDW is a good prognostic marker in patients with HF and DM with respect to the study endpoint. We also showed that RDW longitudinal changes in diabetics differ significantly from the non-diabetic group.

Red blood cell distribution width is a widely available and inexpensive test, which is performed as part of the complete blood count. Previous studies have shown that higher levels of RDW were associated with poor outcome in a number of pathologic states such as HF, coronary artery disease and metabolic syndrome but also in the general population. The presence of comorbidities in patients with HF is not unusual. Among them, DM is a common disorder in HF patients. To our knowledge, data on prognostic value of RDW in patients with DM and HF are scarce.

### RDW and diabetic status

Based on the current bibliography, RDW is a marker of inflammation and prognosis in patients with DM [29, 30]. The association between DM and RDW has been initially examined by Subharshree [31] in a cross sectional study, which showed that BNP correlated well with RDW in diabetic patients with HF. Engström et al. [30] showed that low RDW was associated with a markedly increased risk of developing DM. Low RDW was also related to higher waist circumference, glucose, insulin and triglyceride concentrations. On the contrary, RDW was significantly and positively associated with HbA1c, corresponding an increase in HbA1c of 0.10% per 1 SD increase in RDW. Malandrino et al. [35] investigated the relationship between RDW and DM complications (microvascular and macrovascular) in a population of 2.497 diabetics and concluded that higher RDW values were associated with increased probability of developing vascular complications, HF, myocardial infarction (MI), stroke and nephropathy. Acosta et al. [36] reported shortened half-life of red blood cell in DM.

It is well established that the RDW values become elevated under conditions of increased red cell destruction or ineffective red cell production [1]. Nevertheless, the pathophysiological path of increased RDW in HF syndrome remains unknown. A number of potential mechanisms have been proposed such as nutritional deficiency (vitamin B12, ferrum or folic acid), bone marrow depression or inflammation [1, 37] leading to red blood cells lifespan extension, as a homeostatic adaptation [38]. Thus, in HF, RDW is considered a prognostic marker, which may reflect an underlying inflammatory process [39]. Importantly, inflammation is a common finding in patients with DM [40, 41] and that is probably why DM is called “proinflammatory state” [42]. Notably, in our study HF patients with DM manifested significant higher values of ferritin (which is considered as an acute phase protein of inflammation) [43] and WBC versus those without DM. Sherif et al. [44] suggested that RDW could be used as a marker of inflammation in type 2 DM. On top of this, our hypothesis was that patients with concomitant HF and DM would have different patterns of RDW changes against those with HF but without DM due to higher inflammatory burden. Inflammatory conditions, such as DM, due to multiple pathophysiological mechanisms lead to red blood cells deformation [43]. In particular, the latter lose their typical discoid shape and membrane elasticity [45].

The undesirable effects of high glucose levels are reflected in erythrocytes in multiple ways such as rearrangement of erythrocyte membranes, defects in Hb oxygen binding activity, alterations of mechanical features of the membrane and general aspects of the cell as well [46, 47]. In particular, RBCs in DM patients manifest a rigid membrane with decreased deformability, increased osmotic fragility and enhanced aggregation. In addition, the impaired Na+/K+-ATPase activity leads to electrolyte disturbances, resulting in an increase in the cell size and increased osmotic fragility which, in turn, contributes to the development of microvascular complications. Furthermore, microscopic examination and state of the art technological measurements of the RBC’s in DM patients reveal augmented aggregate shape and size when compared to healthy controls and reduced deformability. The latter results in increased blood viscosity and probable increase in shear stress on the endothelial wall [47, 48].

Pretorius et al. have shown not only that RBCs in type II DM have an altered shape (lose their normal discoid shape and adopt a skewed morphology), and a decreased membrane roughness [45, 49], but also, that they can promptly adapt in a changed environment, including during an addition of glucose to healthy RBCs [49, 50]. The same authors have interestingly reported that the axial ratio of the RBCs from DM patients was significantly greater against that of matched controls, as seemed to occur in a variety of inflammatory diseases [49, 51–53] and raise the question of the probable prognostic and diagnostic significance of these findings. Similarly, Berndt-Zipfel et al., showed that RBC deformability was also altered in type II DM, and that an improved RBC deformability correlated with improved glycaemic control [54]. Apart from RBC’s structure, a number of studies suggest that RBC rheology is also altered in type II DM [49, 55, 56].

Recently, Nigra et al. [57] reported that hyperglycaemia affected the human RBC’s by altering the content and distributions of three tubulin isotypes, resulted in reduction of erythrocyte deformability and osmotic resistance. Although, tubulin is not a major protein by volume in RBC’s, it plays a significant structural role and regulates the enzymatic activity such as Na+/K+-ATPase and PMCA [58, 59]. The findings by Nigra et al., suggest that high glucose concentrations promote tubulin acetylation and its translocation to the membrane, and that this tubulin is involved in regulation of RBC’s deformability and osmotic fragility. This interplay among inflammation and unfavorable effects of high glucose levels on circulating erythrocytes shape, size and mechanical features can have an impact on RDW values. Longitudinal changes of RDW in the group of diabetics were significantly different compared to the non-diabetic group suggesting that inflammation may play a pivotal role in RDW changes. In summary, although RDW can be used for the risk stratification of HF patients with or without DM, it shows different variation patterns with time when we compare these groups.

The presence of anemia is associated with a special risk in patients with any form of proatherosclerotic condition and heart disease [60]. Despite the fact that clinical and hemodynamical changes due to acute, short-lasting anemia are reversible, chronic anemia can lead to cardiac enlargement and left ventricular hypertrophy due to volume overload [61]. Anemia is considered to be a mediator and a marker of a poor outcome in HF [60, 62], a risk factor for ischemic heart disease, and often coexists in patients with acute coronary syndromes [63]. Notably, in patients with stable coronary artery disease, higher RDW is a strong and independent prognostic marker of mortality [8]. In our study, RDW was an independent, to anemia, prognostic factor for death or rehospitalization.

#### Study limitations

The sample size of diabetic patients that enrolled in the study was not large (71 patients), albeit every patient not met the study endpoint, had five measurements (admission, discharge, 4, 8 and 12 months). This means that the results should be interpreted under caution and used as a basis for larger studies.

### Future directions

Subclinical chronic inflammation is a common feature in the natural course of diabetes and levels of inflammatory biomarkers show promising results regarding this correlation [64]. We strongly believe that the design of larger studies with main focus on “specific” markers of inflammation (such as Leptin, TNF, IL-6, CCL2, Resistin, Adiponectin) [65, 66] and the association with RDW would be of particular importance.

## Conclusions

Red blood cell distribution width is an easy to measure, widely available and low cost marker that showed in our study, similar prognostic significance both in the diabetic and non-diabetic group of HF patients with respect to the combined outcome of death from any cause or hospitalization for HF. The RDW longitudinal changes showed significant statistical difference in diabetic and non-diabetic patients. These findings might be due to the higher inflammatory burden that patients with concomitant DM and HF may carry against the population of HF without DM and may provide new insights to the understanding of the pathophysiological mechanism of RDW increase in HF and other pathological states, which remain unclear.

## Abbreviations

RDW: red blood cell distribution width
HF: heart failure
AHF: acute heart failure
DM: diabetes mellitus
